# Combination of high-frequency ultrasound and virtual touch tissue imaging and quantification improve the diagnostic efficiency for mild carpal tunnel syndrome

**DOI:** 10.1186/s12891-021-03982-7

**Published:** 2021-01-26

**Authors:** Zhen-han Lai, Shu-ping Yang, Hao-lin Shen, Yi Luo, Xiao-han Cai, Wen-ting Jiang, Li-ping Liao, Kun-bin Wu, Guo-rong Lv

**Affiliations:** 1 Department of Ultrasound, Zhangzhou Hospital Affiliated to Fujian Medical University, Zhangzhou, 363000 Fujian China; 2 Collaborative Innovation Center for Maternal and Infant Health Service Application Technology, Quanzhou, 362000 Fujian China; 3 Department of Ultrasound, Second Affiliated Hospital of Fujian Medical University, Quanzhou, 362000 Fujian China

**Keywords:** High-frequency ultrasound, Virtual touch tissue imaging and quantification, Carpal tunnel syndrome

## Abstract

**Background:**

Carpal tunnel syndrome (CTS) is the most common entrapment symptom in the peripheral nerves. High-frequency ultrasound (HFUS) is widely used in the diagnosis of CTS. Virtual Touch Tissue Imaging and Quantification (VTIQ), which provides more information about the hardness of organization, is used to diagnose CTS. However, the data of diagnostic value of them in various degrees of CTS are limited. Whether the combination of HFUS and VTIQ can improve the diagnostic efficiency also remains unknown. The study aimed to explore the diagnostic value of HFUS and VTIQ in various degrees of CTS and whether combination of HFUS and VTIQ could improve the diagnostic efficiency of CTS.

**Methods:**

A collection and analysis of 133 CTS patients and 35 volunteers from January 2016 to January 2019 were performed. We compared the clinical characteristics, cross-sectional area (CSA) value and shear wave velocity SWV_mean_ value of CTS group with volunteer group.

**Results:**

The CSA value and SWV_mean_ value of CTS cohort were significantly higher than volunteer group (10.79 ± 2.88 vs. 8.06 ± 1.39, *p* < 0.001, 4.36 ± 0.95 vs. 3.38 ± 1.09, *p <* 0.001, respectively). The area under the curve (AUC) of receiver operating characteristic (ROC) curve of CSA value and SWV_mean_ value were 0.794 and 0.757, respectively. Hierarchical analysis of CSA value and SWV_mean_ value showed that the AUC in the moderate and severe CTS group were higher than in mild CTS group. Furthermore, the CSA value combined with SWV_mean_ value used to diagnose mild CTS was 0.758, which was higher than that of single CSA value or single SWV_mean_ value.

**Conclusions:**

Both HFUS and VTIQ technology were feasible to evaluate CTS. HFUS was suitable for use in diagnosis of moderate and severe CTS. For mild CTS, combination of HFUS and VTIQ was relevant to improve the diagnostic efficiency of CTS.

## Background

Carpal tunnel syndrome (CTS) is a common entrapment neuropathy of the median nerve characterized by paresthesias and pain in the first to fourth digits. The differentiations of anatomical variations, such as those of the palmaris longus (accessory tendons, “reversed” palmaris longus, palmaris profundus muscle), persistent median artery, “bifid” median nerve and others, are a prerequisite condition for the development of CTS [1]. The diagnosis of carpal tunnel syndrome is mostly based on a combination of clinical symptoms, signs, and an imaging examination. Medical ultrasound, an imaging modality, is more and more popular in the diagnosis of CTS [2, 3].

In recent years, ultrasound has been used in many fields since it provided supplement diagnostic guidance, such as parotids gland [4], central lung lesions [5], prostate cancer [6], as well as CTS [7]. Fowler et al [8] has reported that the composite sensitivity and specificity of ultrasound for the diagnosis of CTS were 77.6 and 86.8%, respectively, in a meta-analysis. Kapuścińska et al [9] also found that ultrasound imaging with the use of high-frequency ultrasound (HFUS) was a valuable diagnostic tool for assessing the surgical treatment in CTS patients. The ultrasound examination is a subjective method which depends on the expertise of the physician. Therefore, more and more research reported that newer ultrasound techniques, such as power Doppler, microvascular imaging, and elastography, might improve the specificity and sensitivity of ultrasound examination in the evaluation of CTS [2, 10, 11].

Virtual Touch Tissue Imaging and Quantification (VTIQ) is a kind of shear wave elastography (SWE) with wider range and smaller region of interest (ROI) of sampling frame than other detection methods [12, 13]. Zhang et al [14] reported that median nerve SWV at the carpal tunnel inlet was significantly higher than patients with CTS, which indicated that VTIQ appeared to be a highly reproducible diagnostic technique. Therefore, VTIQ provides more objective and directional information about the hardness of organization and reflects the difference in mechanical properties between inner organizational structure.

Although HFUS and VTIQ have been used to diagnosis CTS in previous studies, the data of diagnostic value of them in various degrees of CTS are limited. Whether the combination of HFUS and VTIQ can improve the diagnostic efficiency also remains unknown. Therefore, the purpose of this study was to explore the diagnostic value of HFUS and VTIQ in various degrees of CTS and whether combination of HFUS and VTIQ could improve the diagnostic efficiency of CTS.

## Methods

### Subjects

This study was performed at the Department of Ultrasound, Zhangzhou Affiliated Hospital of Fujian Medical University. Between January 2016 and January 2019, we collected a total of 133 patients of CTS and 35 healthy volunteers. The diagnostic criteria of CTS were according to the American Academy of Neurology, which included clinical history, symptoms, and evidence of slowing of distal median nerve conduction [15, 16]. The inclusion criteria were as follows: (1) Numbness in the three fingers of the radial side, (2) A history of numbness at night, (3) Atrophy of the thenar muscles, (4) The clinical manifestations and auxiliary examinations of the patients all conformed to different degrees of carpal tunnel syndrome, (5) Actively cooperate with treatment and postoperative follow-up. All CTS patients met all the above inclusion criteria. The exclusion criteria were as follows: (1) history of wrist trauma surgery, (2) space-occupying lesions in the carpal canal, (3) combination with other peripheral nerve diseases, such as thoracic outlet syndrome, cubital tunnel syndrome, and carpal ulnar tunnel syndrome, (4) lost in following up. We further divided CTS into mild, moderate, and severe stage, according to criteria as previous reported [17]. The control group included 35 healthy volunteers, they did not fulfill any of the following criteria: (1) Numbness in the three fingers of the radial side, (2) A history of numbness at night, (3) Atrophy of the thenar muscles, (4) Other disorders such as upper limb trauma, hypertension, diabetes, hyperthyroidism, or hypothyroidism. They received the same ultrasound evaluation protocol as provided for CTS patients. All patients and healthy volunteers provided informed written consent. The ethics committee of our hospital approved this study (no.2018-LX-015).

### Electrodiagnostic examinations

Electrodiagnostic examinations were performed according to the protocol suggested by Li et al [18] for CTS patients. The four-way EMG induction potentiometer of KEYPOINT 4 from Denmark was used in this study.

### High-frequency ultrasound diagnosis (HFUS)

**Fig. 1:**
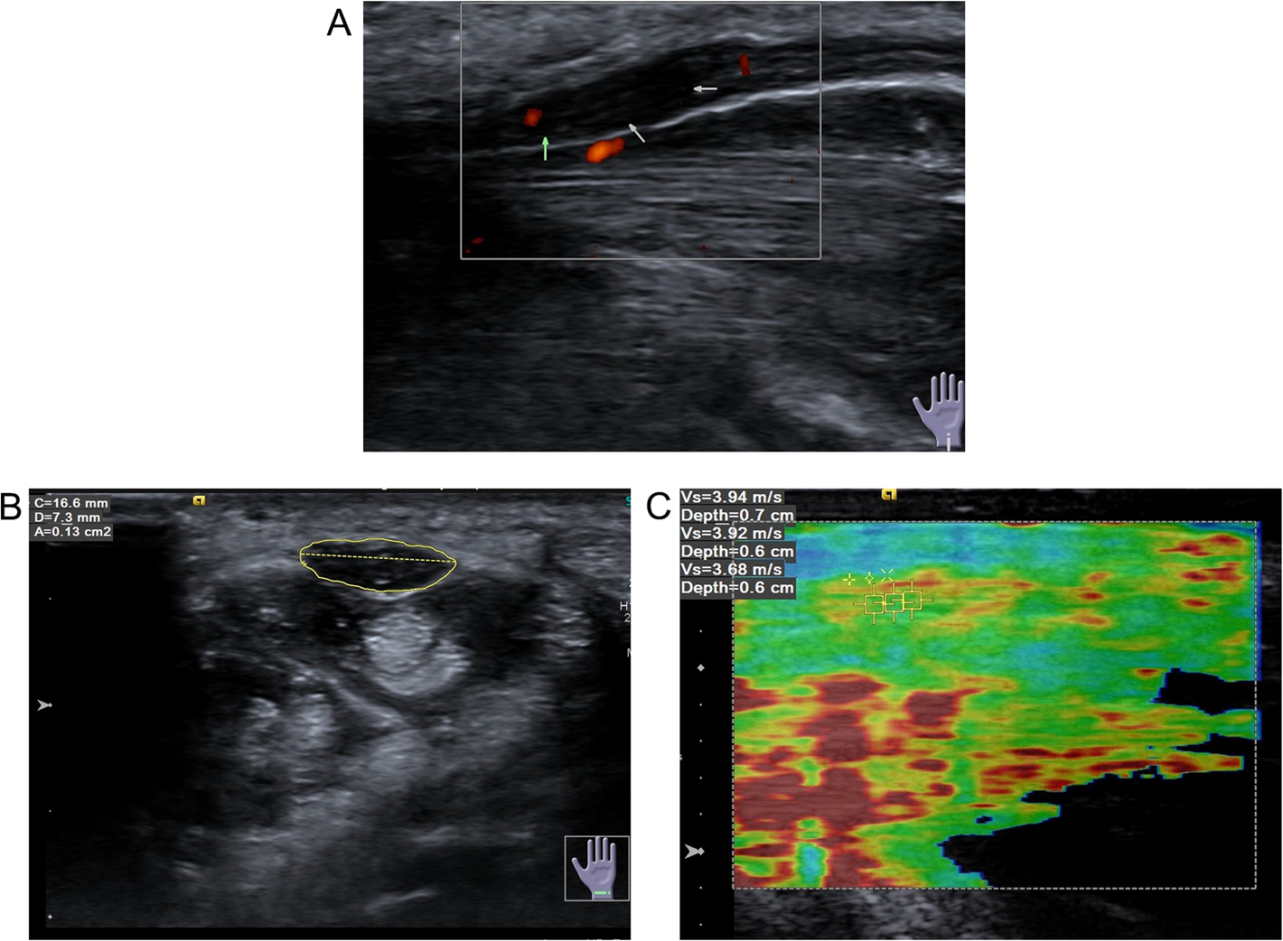
Ultrasound diagnosis (**a**) The longitudinal section of HFUS showed thickening of the middle normal nerve in carpal canal, decreased echo, and increased blood flow signal in power Doppler ultrasound; **b** Median nerve CSA was measured using the pisiform bone as a reference; **c** ROI was measured three times at the carpal tunnel inlet and outlet parallel to the pisiform bone and groove bone and different areas in the median nerve at the midpoint of the carpal canal

Ultrasound imaging was performed using a 9 L-4 probe and a standard 4–9 MHz transducer (Acuson S3000; Simens AG). Participants were seated with forearm supinated, the wrist resting on a flat surface in a neutral position and palm up. Enough amount of coupling agent was used to the skin. The probe was positioned perpendicular to the surface of the median nerve gently without any pressure. The median nerve was scanned from the cross-section of the forearm from the socket of the elbow to the wrist for showing the cross-section of the median nerve. The transducer was rotated 90 ° to scan along the long axis of the nerve (Fig. 1a). The median nerve cross-sectional area (CSA) was measured by the pisiform bone as a reference (Fig. 1b). Each participant was examined three times by the same radiologist, and the mean value was the outcome.

### Virtual touch tissue imaging quantification (VTIQ)

An image was generated from using a 4–9 MHz and 9 L-4 probe combined with VTIQ. The radiologist placed the transducer on the carpal tunnel surface parallel to the median nerve. The probe was fixed respectively at the carpal tunnel inlet and outlet parallel to the pisiform bone and groove bone. The quality control model was used to evaluate the quality of the shear wave. In the image, a high-quality area was labeled green, otherwise bad quality. ROI was replaced at the good quality area. Then we switched the velocity mode in the high-quality area and measured the SWV of the median nerve. The minimum range of ROI was 1mm^2^ (multiplying 1 mm by 1 mm). ROI was measured three times at the carpal tunnel inlet, and outlet parallel to the pisiform bone and groove bone and different areas in the median nerve at the midpoint of the carpal canal (Fig. 1c). All parameters were measured by the double-blind method. As a result, the mean value (V mean) was calculated, namely, SWV_mean_.

### Statistical analysis

All data analyses were performed by SPSS software (IBM Corp. Released in 2015. IBM SPSS Statistics for Windows, Version 23.0. Armonk, NY: IBM Corp.). The data were presented as the mean ± standard deviation (SD) for continuous variables and as a number for categorical variables. Chi-squared test or Fisher’s exact test was used to compare the categorical variables. Comparisons between CTS group and volunteer group were made for continuous variables using Student’s *t* test. One-way ANOVA was used to compare the continuous variables between groups stratified by CTS severity. Statistical significant was defined as *p* value less than 0.05.

## Results

### Clinical characteristics

The clinical characteristics of the 168 subjects (133 CTS and 35 volunteers) were listed in Table 1. The CSA value of the CTS cohort was significantly higher than volunteers (10.79 ± 2.88 vs. 8.06 ± 1.39, *p* < 0.001), while the SWV_mean_ value also significantly higher than volunteers (4.36 ± 0.95 vs. 3.38 ± 1.09, *p* < 0.001). But there were no significant differences in age, gender, and location between the two groups (*p* > 0.05). Demographic and clinical variables of patients with mild, moderate, and severe CTS were summarized in Table 2. The CSA and SWV_mean_ value increased significantly with the aggravation of the disease severity. There is no statistically difference in other parameters among three groups.

**Table 1:** Demographic and clinical variables according to the disease status of CTS

Characteristic	CTS (*n* = 133)	Volunteer (*n* = 35) (*n* = 1540)	P
Age [year, mean (SD)]	51.79 (12.94)	52.60 (11.72)	0.937
Gender (%)			
Male	32 (24.1)	10 (28.6)	0.661
Female	101 (75.9)	25 (71.4)	
Location (%)			0.851
Left hand	71 (53.4)	18 (51.4)	
Right hand	62 (46.6)	17 (48.6)	
CSA^a^ [mm^2^, mean (SD)]	10.79 (2.88)	8.06 (1.39)	< 0.001
SWV^b^ mean [m/s, mean (SD)]	4.36 (0.95)	3.38 (1.09)	< 0.001

^a^cross-sectional area

^b^shear wave velocity

**Table 2:** Demographic and clinical variables of patients with mild, moderate, and severe CTS

Clinical classification	Mild (*n* = 36)	Moderate (*n* = 46)	Severe (*n* = 51) (*n =* 1540)	P
Age [year, mean (SD)]	50.47 (15.04)	54.22 (12.51)	52.55 (11.63)	0.431
Gender (%)				0.394
Male	26 (72.2)	33 (71.7)	42 (31.6)	
Female	10 (27.8)	13 (28.3)	9 (17.6)	
Location (%)				0.390
Left hand	17 (47.2)	23 (50.0)	31 (60.8)	
Right hand	19 (52.8)	23 (50.0)	20 (39.2)	
CSA^a^ [mm^2^, mean (SD)]	9.03 (1.95)	10.35 (2.25)	12.43 (3.07)	<0.001
SWV^b^ mean [m/s, mean (SD)]	3.92 (0.67)	4.38 (1.09)	4.64 (0.88)	0.002

^a^cross-sectional area

^b^shear wave velocity

### Diagnostic efficiency of HFUS combined VTIQ for mild-CTS

**Fig. 2:**
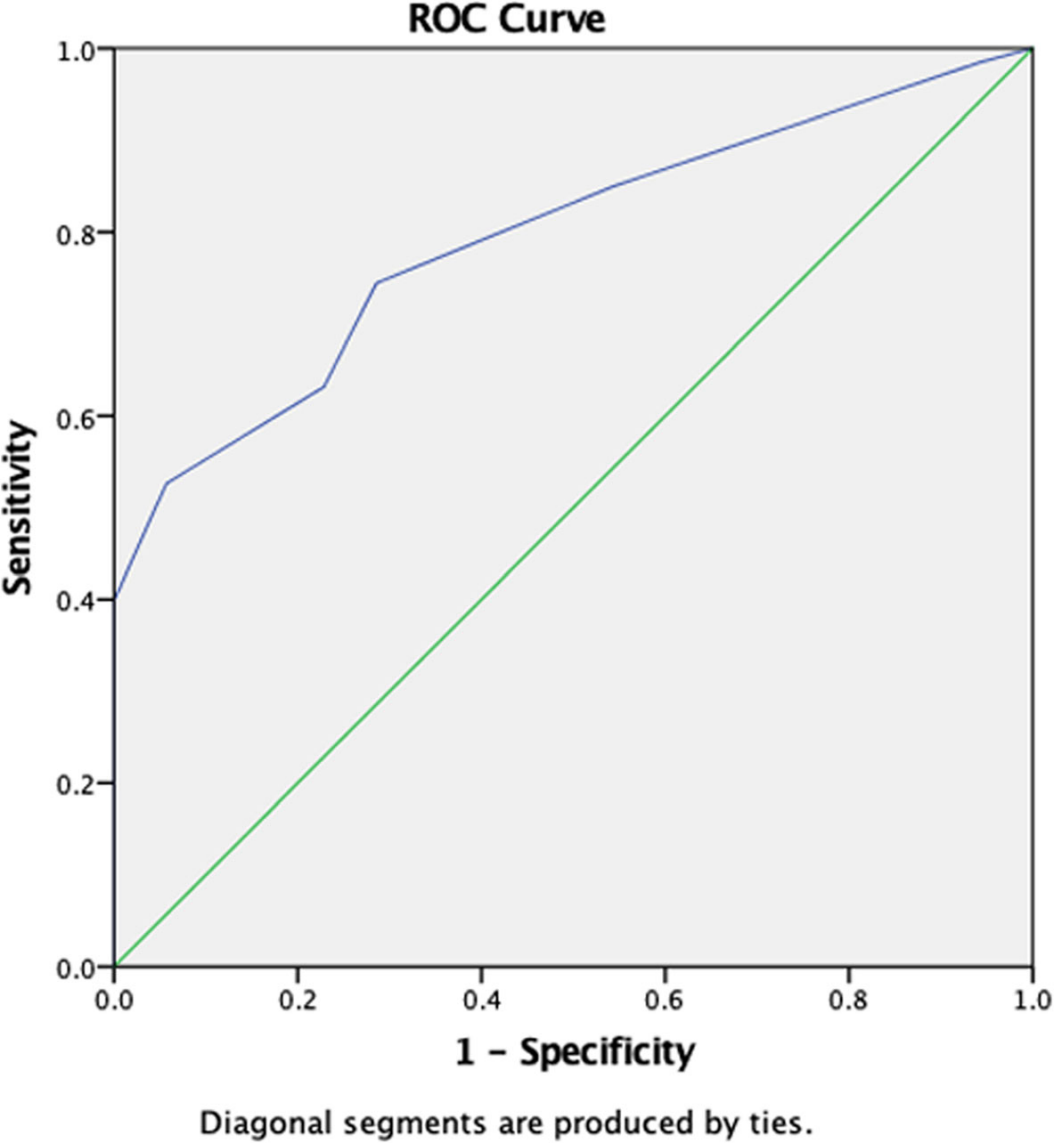
ROC curve for CSA value in diagnosing CTS

**Fig. 3:**
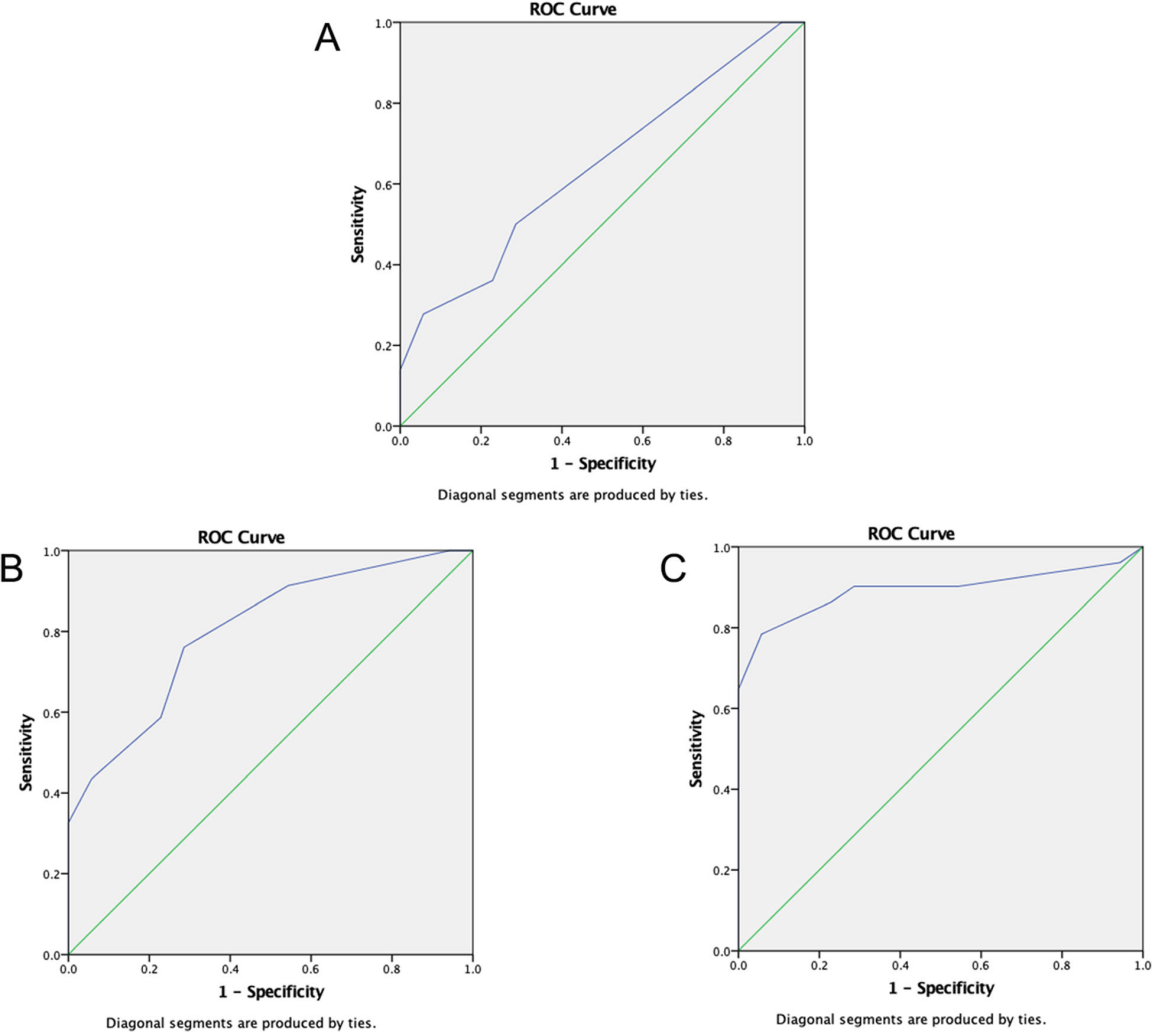
ROC curve for CSA value in diagnosing different degree of CTS (**a**. mild CTS; **b** moderate CTS; **c** severe CTS)

We initially drew receiver operating characteristic (ROC) curves for CSA value in diagnosing CTS (Fig. 2). Youden index was calculated to acquire the optimum threshold value. The sensitivity, specificity and accuracy were used to evaluate the diagnostic efficiency. 8.50 mm^2^ (AUC, 0.794; 95% confidence interval, 0.723–0.864), the threshold value of the CSA for diagnosing CTS by HFUS, the sensitivity, specificity and accuracy were 74.4, 71.4 and 79.4%, respectively. According to CTS stages, we performed hierarchical analysis of CSA based on different degrees (Fig. 3). The results showed that the AUC in moderate and severe CTS group were respectively 0.803 and 0.893, which were significantly higher than mild CTS (AUC, 0.641). Therefore, HFUS had a better performance in diagnosis of moderate or severe CTS. However, diagnostic efficiency of HFUS should be further improved for mild CTS patients.

**Fig. 4:**
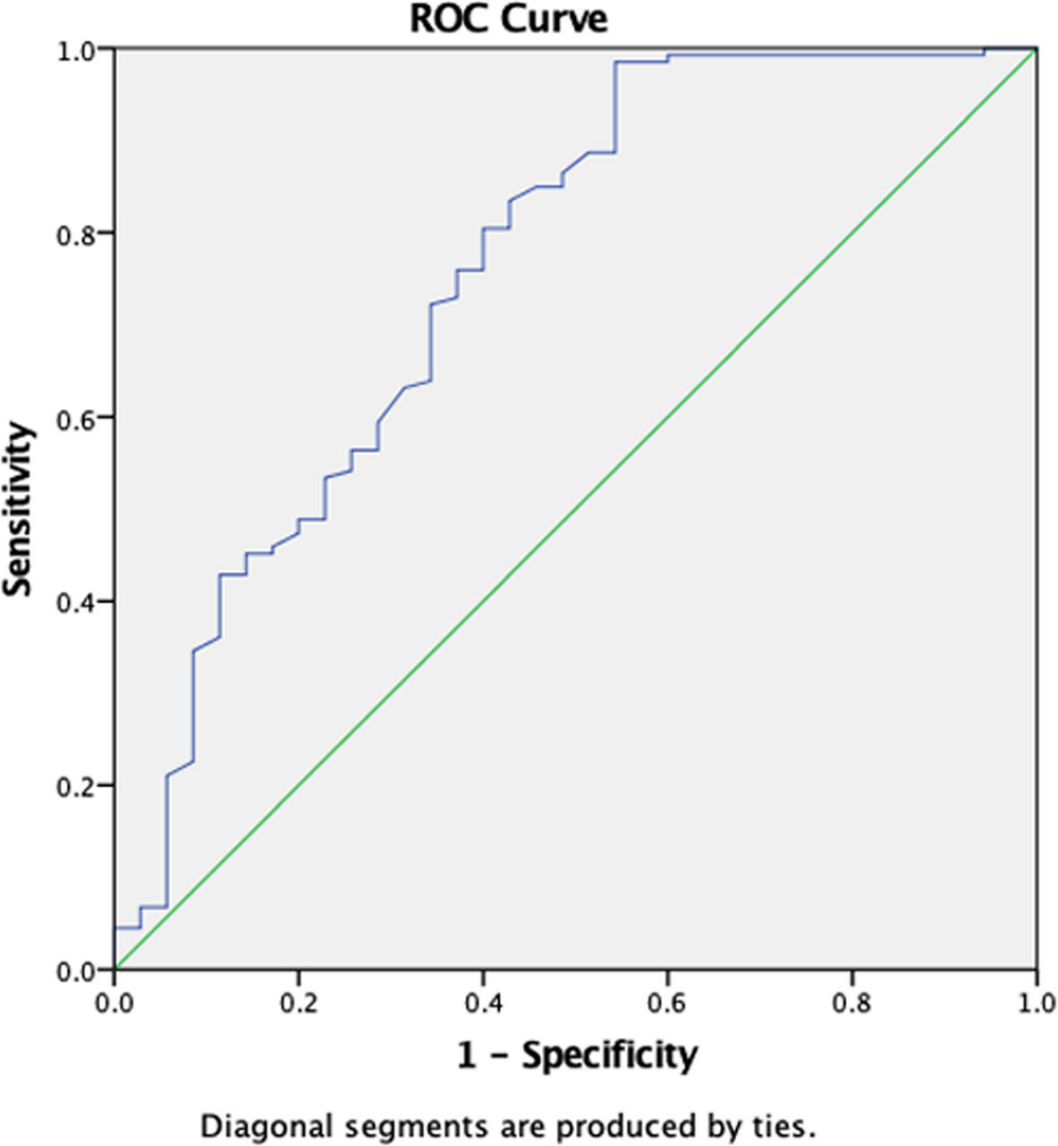
ROC curve for SWV_mean_ value in diagnosing CTS

**Fig. 5:**
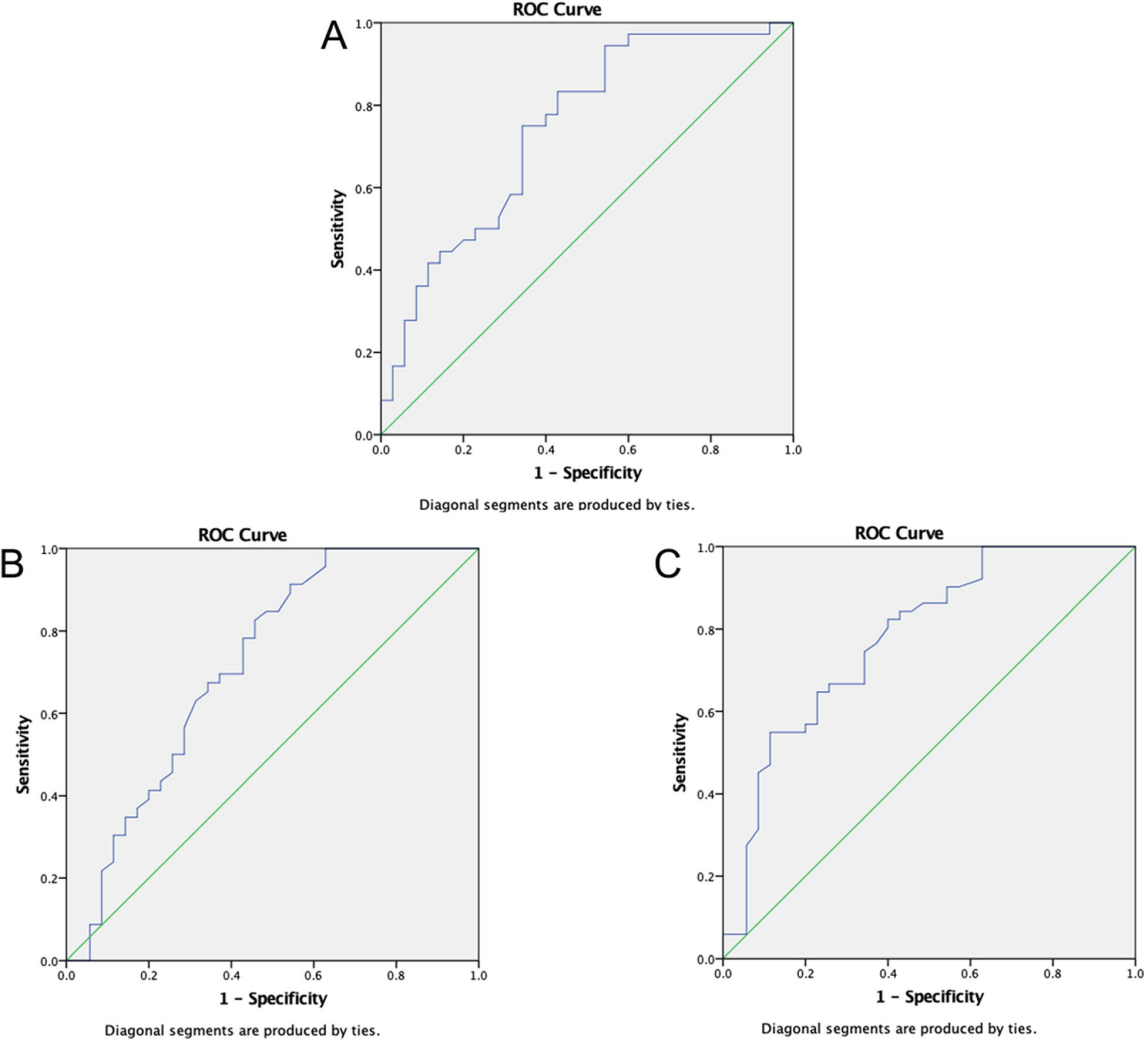
ROC curve for SWV_mean_ value in diagnosing different degree of CTS (**a**. mild CTS; **b** moderate CTS; **c** severe CTS)

**Fig. 6:**
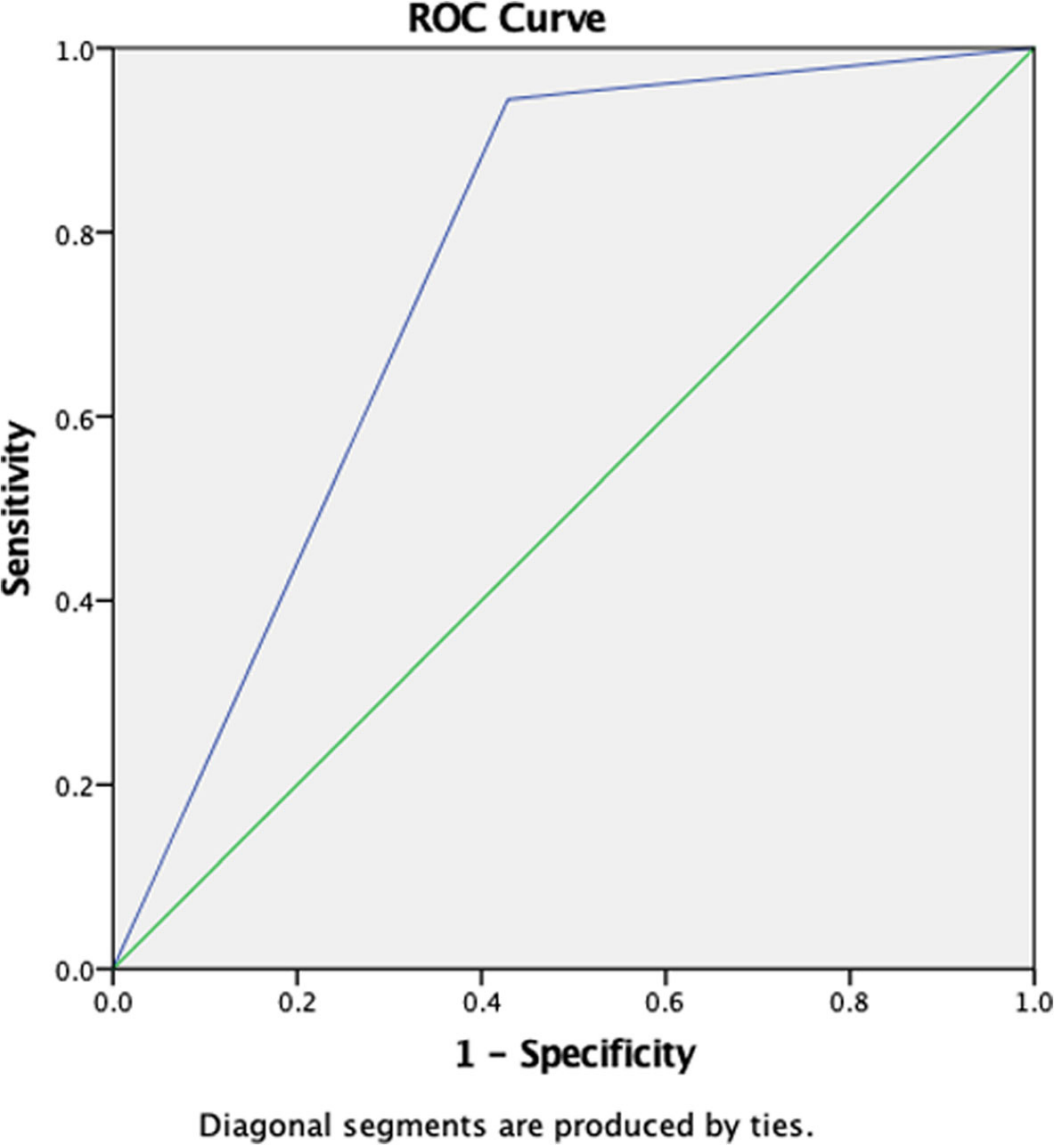
ROC curve for CSA combined with SWV_mean_ value in diagnosing mild CTS

We performed ROC curve for SWV_mean_ value in diagnosing CTS (Fig. 4). The curve graph showed that the AUC was 0.757. According to the various degrees of CTS, we performed hierarchical analysis of SWV_mean_ value (Fig. 5). Hierarchical analysis suggested that the AUC in mild, moderate, and severe CTS group were 0.742, 0.718 and 0.778 respectively. The results revealed that the diagnostic efficiency of HFUS for mild CTS was lower than that of VTIQ. But the diagnostic efficiency of HFUS for moderate and severe CTS was higher than that of VTIQ. Furthermore, we conducted a ROC curve for CSA combined with SWV_mean_ value in diagnosing mild CTS (Fig. 6). We found that the AUC was 0.758. The sensitivity, specificity and accuracy were 94.4, 57.1, and 75.8% respectively. Hence, combination of HFUS and VTIQ can improve the diagnostic efficiency of mild CTS.

## Discussion

CTS is the most common entrapment neuropathy and it is caused by compression of the median nerve at the wrist. It is a clinical syndrome characterized by paresthesia and dysfunction in the corresponding innervated area due to the compression of median nerve in the carpal canal [19]. In the past, the diagnosis of carpal tunnel syndrome largely depends on clinical manifestations and electromyography. With the development of ultrasound technology, HFUS can clearly show the morphological changes of the median nerve in the carpal canal and the anatomical relationship with the surrounding tissues so that the location and causes of the median nerve compression in the wrist can be clarified [20].

Our study found that the CSA value was 10.79 ± 2.88 mm^2^ in CTS cohort, which was significantly higher than in volunteers. In previous report [21, 22], the median nerve abnormality CSA was higher than 10 mm^2^, which was consistent with our findings. HFUS was considered to have the highest diagnostic value in evaluating median nerve CSA because the location of median nerve at the level of pisiform bone is superficial, easy to display, and the swelling was most obvious. According to ROC curve, the optimum threshold value was 8.50 mm^2^ (AUC, 0.794; 95% confidence interval, 0.723–0.864) in our study. At the optimum threshold value, the sensitivity, specificity and accuracy of HFUS were 74.4, 71.4, and 79.4% respectively. These results showed that HFUS had a higher diagnostic efficiency.

However, studies have shown that about 30% of CTS patients, median nerve CSA did not increase and was not associated with the stage of disease, hence it is not enough to diagnose median nerve disease by observing CSA only [23]. According to the degree of CTS, we performed a hierarchical analysis of CSA. (Fig. 3). Results demonstrated that the AUC in moderate and severe CTS group were 0.803 and 0.893 respectively, which was significantly higher than in mild-CTS (AUC, 0.641). HFUS yielded a high diagnostic efficiency in moderate or severe CTS patients, but this was not case in mild CTS patients. Therefore, diagnostic efficiency of mild CTS patients should be further improved.

The SWV of median nerve was measured by VTIQ technology to diagnose CTS, indicating that the nerve hardness of wrist CTS was higher than that of the control group in this study. The SWV_mean_ value of CTS cohort was also significantly higher than volunteers (4.36 ± 0.95 vs. 3.38 ± 1.09, *p* < 0.001). By using the SWE technique, the pathological mechanism of CTS showed that the continuous increase of carpal canal pressure over a long period of time, which affected the circulation of median nerve, leading to a series of nerve membrane edema, fibroblast infiltration and nerve fiber degeneration, and then the median nerve was damaged. Increased median fibrosis and pressure in the carpal canal may lead to increased nerve hardness of CTS [14].

VTIQ can provide more objective and direct information about the hardness of the organization and reflect the difference in mechanical properties within the organization, which is the main reason why this new technology can be used to diagnose CTS. Figure 4 showed that the AUC was 0.757 in ROC for SWV_mean_ value. According to the various severity of CTS, we performed hierarchical analysis of SWV_mean_ value (Fig. 5). Results showed that the AUC in mild, moderate, and severe CTS group were 0.742, 0.718, and 0.778, respectively. The above results suggested that the diagnostic efficiency of HFUS for mild CTS was lower than that of VTIQ. At 2.955 m/s (AUC, 0.742; 95% confidence interval, 0.627–0.857) in this study, which was accepted as the threshold value of the SWV_mean_ for diagnosing mild CTS by VTIQ, the sensitivity, specificity, and accuracy were 94.4, 40, and 74.2%, respectively.

VTIQ technology indirectly provided the soft and hard information of the study object only. There are other factors that affect truly soft and hard degree of the tissue reflected by elastography technology, which isthe possible reason that the diagnostic efficiency of VTIQ and technology on CTS is lower than that of HFUS and technology suggested by the results of this study. Interestingly, we performed a ROC curve for CSA value combined with the SWV_mean_ value of mild CTS. As revealed in the Fig. 6, the curve showed that the AUC was 0.758. The sensitivity, specificity, and accuracy were 94.4, 57.1, and 75.8% respectively. Hence, combination of HFUS and VTIQ can improve the diagnostic efficiency of mild CTS.

We found that the application of VTIQ technology did not improve the diagnostic efficiency of CTS. The reasons may be related to the measurement limitations of VTIQ technology. Firstly, the median nerve was thin and superficial, especially in the carpal canal. The probe needs to be pressed against the skin during operation, which was inevitably affected by external forces and other factors. Secondly, the swelling of the median nerve in the carpal canal and the elastic modulus were different in patients with different degrees of CTS and the early SWE value may not change.

Several limitations of the present study required consideration. First, this is a single-center study. It would be necessary to conduct larger, multi-center studies of the general population in the future. Second, this study had a small sample size, which did not allow for a subgroup or stratified analysis.

To sum up, both HFUS and VTIQ technology were feasible to evaluate CTS. We recommended that HFUS was used to diagnose moderate and severe CTS group, which was more economical than VTIQ. For mild CTS, combination of HFUS and VTIQ is suggested to improve diagnostic efficiency.

## Data Availability

The datasets used and/or analysed during the current study available from the corresponding author on reasonable request.

## Abbreviations

CTS: Carpal Tunnel Syndrome
HFUS: High-Frequency Ultrasound
VTIQ: Virtual Touch Tissue Imaging and Quantification
SWE: Shear Wave Elastography
ROI: Region of Interest
SWV: Shear Wave Velocity
CSA: Cross-Sectional Area
ROC curve: Receiver Operating Characteristic curve
AUC: Area Under Curve
